# The Impact of AI on Learners’ Self-Efficacy: A Meta-Analysis

**DOI:** 10.3390/bs16010158

**Published:** 2026-01-22

**Authors:** Liling Ren, Jason M. Stephens, Kerry Lee

**Affiliations:** Faculty of Arts and Education, School of Education and Social Practice, University of Auckland, Auckland 1010, New Zealand

**Keywords:** artificial intelligence, self-efficacy, meta-analysis, AIED

## Abstract

With the rise of generative artificial intelligence, the application of AI in learning environments has received widespread attention. Although empirical studies have explored the effect of AI on self-efficacy, the results have not been consistent. This study conducted a meta-analysis on the results from 23 empirical studies on the impact of AI use on self-efficacy. These studies were published between January 2005 and February 2025 and indexed in one or more of the three major educational research databases: Web of Science, Scopus, and ERIC. The results indicated that AI had a significant positive impact on self-efficacy in learning contexts (effect size of 0.758). Specifically, discipline (Q = 10.348, *p* < 0.05) and the specific role played by AI (Q = 3.991, *p* < 0.05) significantly moderated the effect of AI on self-efficacy. In our discussion, suggestions are provided for enhancing learner self-efficacy and improving the effectiveness of AI in the learning contexts.

## 1. Background

Artificial intelligence (AI) is an influential technology that is impacting students and educators in all fields at every level (Morales-García et al., 2024). With its potential to improve learning, teaching, and management, AI is sparking widespread attention among scholars interested in its application to education (Ng et al., 2023). When compared to traditional learning contexts, AI-enabled learning environments provide the opportunity to bring richer learning experiences to students (Ren et al., 2025), such as personalized learning support and intelligent learning assistance, which are believed to help improve students’ criticality and creativity (Volante et al., 2023).

Meanwhile, AI has become an increasingly favorable tool for analyzing complicated data (Talaei Khoei et al., 2023) to comprehensively explore students’ learning behavioral patterns (Raihan et al., 2024). In a study conducted by Cerezo et al. (2020), an AI system was used to observe students’ self-regulated learning (SRL) process and collect the research data. AI has been shown to significantly improve students’ self-efficacy through its unique features, such as providing an adaptable learning environment and personalized learning experience (Massaty et al., 2024). In this way, AI has the potential to have an impact on psychological factors that affect students’ learning (J. Wang et al., 2025; Salah et al., 2024).

Self-efficacy refers to an individual’s beliefs about their ability to perform a specific task and plays an important role in influencing human performance (Bandura, 1997; Jeong & Jeong, 2024). Research has shown self-efficacy to be a positive predictor of academic achievement (Shen et al., 2013). Students who hold high self-efficacy exert greater effort and display more persistence than students who doubt their abilities and have low self-efficacy (Schunk, 1990).

In addition, self-efficacy is affected by many elements, such as feedback and rewards, communication and interactions, and motivation and attitude (Peechapol et al., 2018). Numerous AI technologies have the potential to enhance self-efficacy through one or more of these elements. Generative AI (capable of creating new content such as text, images, or code), such as ChatGPT and DeepSeek, can interact with individuals by voice or text in a human manner, enhancing students’ one-on-one communication, engagement, and learning experiences (Labadze et al., 2023). AI-mediated language learning platforms support personalized lessons, which can give real-time feedback to students (Wei, 2023). Studies have already shown the ability of AI to develop self-efficacy. For example, AI adoption in organizations shown to boost creative self-efficacy (Jeong & Jeong, 2024), whilst generative AI has been shown to enhance the self-efficacy and higher-order thinking of preservice teachers (Lu et al., 2024).

A comprehensive understanding of the impact of AI on learners’ self-efficacy in educational contexts is crucial for enhancing students’ learning effectiveness in AI-enabled learning contexts, but there are few reviews that comprehensively explore this area. Although the systematic review of Massaty et al. (2024) explored the impact of AI on self-efficacy, it mainly focused on the aspect of computational thinking. To fill this gap, this study conducts a meta-analysis of the impact of AI on learners’ self-efficacy in educational contexts, aiming to address the following questions:

(1) How effective is AI in promoting learners’ self-efficacy in learning contexts?
(2) How do characteristics such as learner levels, disciplines, the type of AI utilized, research settings, the role of AI, and the duration of the study moderate the influencing effect?

## 2. Methods

### 2.1. Data Sources and Search Strategy

This study employed a meta-analytic methodology to address the inconsistent conclusions regarding the effects of AI on self-efficacy. Specifically, this study adopted a search strategy using three databases, well-known in the field of education (Web of Science, Scopus, and ERIC), to retrieve and download relevant studies from academic journals and conference papers. The inclusion criteria for this study are shown in Table 1.

### 2.2. Literature Search and Screening

In this study, we used Boolean operators (AND, OR and NOT) in these three databases, and the specific search string was (“AI” OR “Artificial intelligence” OR “Machine intelligence” OR “Machine learning” OR “Neural network” OR “Intelligence virtual reality” OR “Intelligent agent” OR “Natural language processing” OR “Recommendation system” OR “Intelligent tutoring system” OR “Expert system” OR “Chatbot”) AND (“self-efficacy”) AND (“education” OR “learning” OR “educational” OR “pedagogical”). All articles were imported into the application Rayyan (Ouzzani et al., 2016) for screening. To ensure strict adherence to the inclusion criteria, the first coder reviewed the screening of all the collected articles, with a second coder screening 25% of all articles. The screening inter-coder consistency was 94.23%, and consensus was reached to resolve any discrepancies.

**Figure 1:**
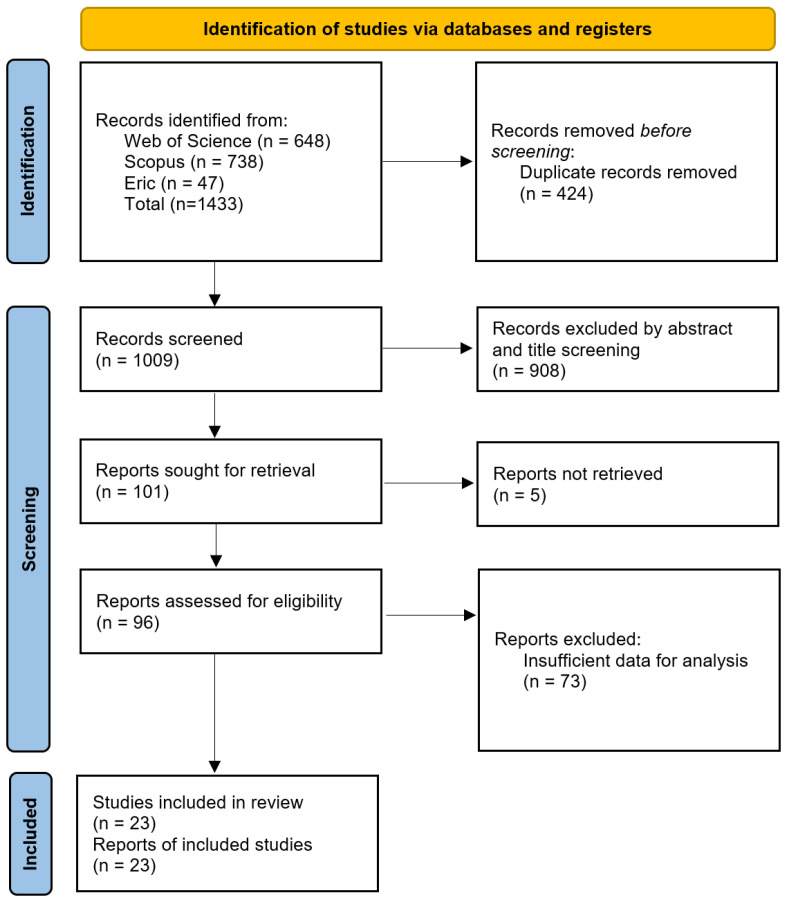
PRISMA flow diagram for the study investigating the impact of AI on learners’ self-efficacy. Note: this figure illustrates how the studies were selected for this meta-analysis. Adapted from the 2021 PRISMA diagram format by Page et al. (2021).

We used a 2021 PRISMA diagram format to describe the search strategy (see Figure 1). We screened for year and study type and downloaded 1433 articles. Duplicates were removed, and then the titles and abstracts were reviewed, which reduced the number of articles down to 101. The articles were reviewed to ensure they met all the established inclusion criteria, and the remaining 96 studies underwent full-text screening. Finally, 23 eligible articles were identified for the meta-analysis.

### 2.3. Coding

In this study, we referred to existing coding strategies (Y. Wang et al., 2024; L. Zheng et al., 2023) to create current coding for the moderating variables; these are, specifically, (1) learner levels: K-12, university, and others; (2) research settings: classroom, online learning, mixed, and others; (3) disciplines: natural sciences, social sciences, engineering, medical, and humanities; (4) type of AI utilized: learning prediction, intelligent tutoring system, student behavior detection, intelligent learning environment, educational robot, and others; (5) the role of AI: intelligent tutor, intelligent learning tool, mixed, and others; and (6) durations of the experiment: <1 month, 1–3 months, >3 months, or not clearly defined. Two coders independently analyzed all 23 articles, and 20 documents had the same coding results. Using the average mutual agreement and reliability formulas, the K value was calculated to be 86.96%, while the reliability coefficient was determined to be 0.93, which indicated that the coding framework demonstrated high reliability (Gaur & Kumar, 2018). Finally, the discrepancies were solved by face-to-face discussion.

### 2.4. Research Quality Assessment

Criteria to assess research quality were derived from previous methodologies (Kmet et al., 2004; Y. Wang et al., 2024). The specific quality assessment criteria for this study are as follows: (1) The question is sufficiently described. (2) Sample characteristics are clearly described. (3) The study design is evident and appropriate. (4) Measurement tools are clearly described. (5) Analytic methods are described and appropriate. Each criterion was ranked according to whether it was deemed clear, relatively clear, and not clear, and was assigned 3 points, 2 points, and 1 point, respectively. Two coders evaluated the articles with a consistency of 91.30%. Each article scored between 11 and 15 points, representing the quality of the literature included in current meta-analysis, which met the requirements.

### 2.5. Statistical Analyses

#### 2.5.1. Effect Size Calculations

Most of our studies had small sample sizes, and for this reason we used Hedges’ g to estimate effect sizes (Hedges & Olkin, 2014). Hedges’ g is a primary measure for the standardized mean difference for studies with small sample sizes (Borenstein et al., 2021). To describe the effect size of this study, we utilized Cohen’s (2013) definitions represented as small (≤0.20), medium (0.20~0.80), and large (≥0.80).

#### 2.5.2. Analyses of Heterogeneity

This study utilized a comprehensive meta-analysis 3.0 (CMA 3.0) approach (J. Chen et al., 2022). We used the Q-test with the I^2^-test to calculate heterogeneity, and the results showed that Q = 213.115, *p* < 0.05, I^2^ = 89.677 (see first table in Section 3), and the I^2^ values were greater than 75%, indicating strong heterogeneity between studies (Higgins et al., 2003). Therefore, the random-effects model was used for the meta-analysis of this study. This indicates that there may be potential moderating variables for the effect of AI on self-efficacy; thus, we conducted a moderating variable analysis.

#### 2.5.3. Publication Bias and Sensitive Analysis

**Figure 2:**
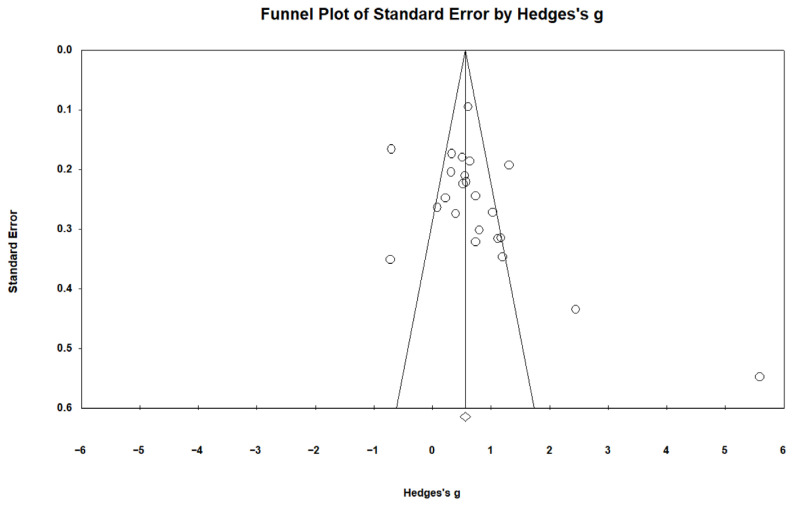
The funnel plot by Hedge’s g (random effects).

This study adopted a funnel plot, classic fail-safe N and Egger’s test to assess the publication bias. The effect sizes shown in the funnel plot are evenly distributed on either side of the summed effect size, which provides a preliminary indication that there is no serious publication bias in the selected studies (J. Chen et al., 2022); see Figure 2. The results of the classic fail-safe N provided a Z-value = 13.606, *p* < 0.001, which indicated we needed to include over 1086 unpublished articles to decease the overall effect. The number of articles (i.e., 1086) was over 125, based on the test standard 5k + 10, where k represents the 23 studies included in this meta-analysis (Rosenthal, 1995). This indicates the results of the meta-analysis of this study are robust and are not affected by a substantial publication bias. The result of an Egger’s test did not attain statistical significance (*p* = 0.073 > 0.05) (Egger et al., 1997), also suggesting the absence of substantial publication bias (F. Wang et al., 2024). The above multiple validation results indicated that there is no substantial publication bias in this paper, and the validity of the sample included in the meta-analysis was high.

**Figure 3:**
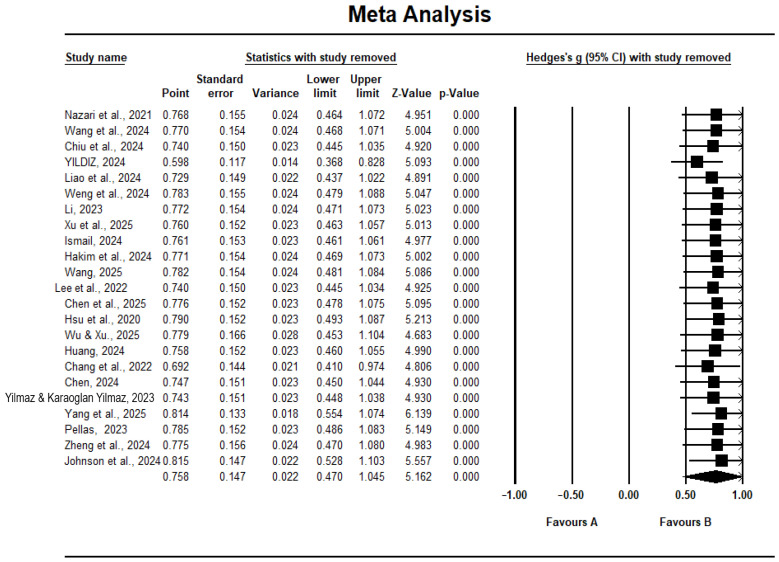
The results of the sensitivity analysis. Note: The data sources are from (Chang et al., 2022; M. A. Chen, 2024; Chiu et al., 2024; A. Chen et al., 2025; Hakim et al., 2024; Hsu et al., 2020; Huang, 2024; Ismail, 2024; Johnson et al., 2024; Lee et al., 2022; Li, 2023; Liao et al., 2024; Nazari et al., 2021; Pellas, 2023; D. Wang et al., 2024; S. Wang, 2025; Weng et al., 2024; Wu & Xu, 2025; Xu et al., 2025; Yang et al., 2025; Yilmaz & Karaoglan Yilmaz, 2023; Yıldız, 2024; R. Zheng et al., 2024).

We also used the one-study-removed method (J. Chen et al., 2022) to perform sensitivity analysis to test anomalous data and robustness. The result showed that effect sizes were relatively stable regardless of which study was deleted. Specifically, a random effects model was used, with effects values biased in the range [0.598, 0.815], indicating that the result is reliable (see Figure 3).

## 3. Results

### 3.1. Overall Effect Size of AI on Self-Efficacy

Table 2 shows the meta-analysis results for the overall effect size of AI on self-efficacy; specifically, the total effect size is 0.758, and the 95% confidence intervals range from 0.470 to 1.045, revealing a medium effect size according to Cohen’s (2013) suggestion (i.e., medium effect range from 0.20 to 0.80).

**Figure 4:**
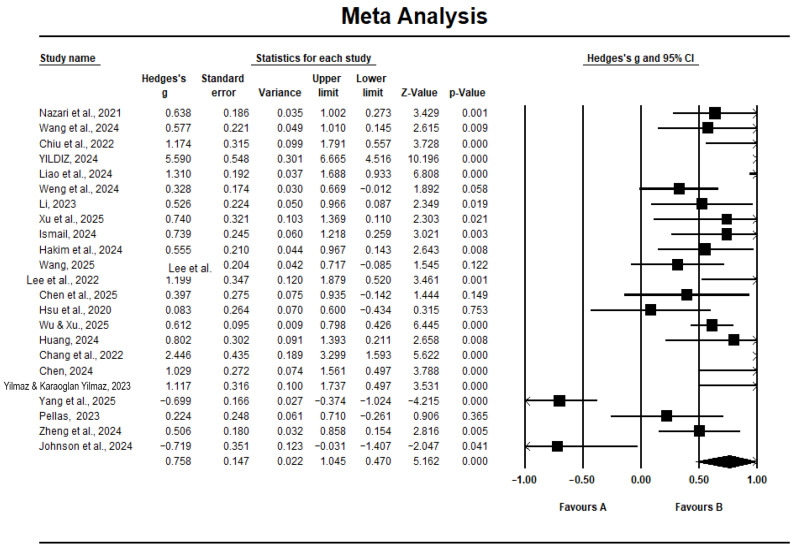
Forest plot. Note: see reference in Figure 3 or reference list for author details.

In Figure 4, the forest plot shows the effect sizes versus weights for each study separately. Among the selected studies, 16 effect values have positive and significant effects, 2 items have negative and significant effects, and the other 5 effect values were not significant.

### 3.2. Moderator Analysis

#### 3.2.1. Learner Levels

As shown in Table 3, the overall composite effect size for learner levels was 0.745, *p* < 0.05. The effect size for university students was 0.813, *p* < 0.05, while the effects for K-12 and other learners were not significant. Overall, there was no significant difference in the effect of AI on the self-efficacy of learners at different levels of study by the results measured of the group effect (Q = 2.740, *p* = 0.254).

#### 3.2.2. Research Settings

As shown in Table 4, the overall composite effect size for different research settings was 0.715, *p* < 0.05. However, there was no significant difference in the effect of AI on learners’ self-efficacy in different research settings, as shown by the results of the test of group effect (Q = 0.289, *p* = 0.591).

#### 3.2.3. Disciplines

There was a significant difference in the effect of AI on self-efficacy across disciplines (Q = 10.348, *p* = 0.035) (see Table 5). Specifically, AI had a high effect value on learner self-efficacy in natural science subjects (1.310, *p* < 0.05) as well as medicine subjects (1.013, *p* < 0.05), while it was not significant for learner self-efficacy in engineering subjects (*p* = 0.894).

#### 3.2.4. Type of AI Utilized

The overall composite effect size for type of AI utilized reached 0.574 (*p* < 0.05), and the effect sizes for each type within the group were significant. However, as can be seen in Table 6, there was no significant difference between AI on learners’ self-efficacy in terms of type of AI utilized (Q = 5.392, *p* = 0.067).

#### 3.2.5. The Role of AI

As shown in Table 7, the overall composite effect size for the role of AI was 0.544, *p* < 0.05, while different AI application roles were found to have significant differences on learners’ self-efficacy (Q = 3.991, *p* < 0.05). The role of AI as an intelligent learning tool had the highest effect value on learner self-efficacy (0.883, *p* < 0.05), while as a mixed tool (i.e., both for intelligent learning tools and intelligent tutors) it had a relatively low effect value on learner self-efficacy (0.450, *p* < 0.05).

#### 3.2.6. Duration of Studies

The overall composite effect size for the duration of the studies reached 0.667 (*p* < 0.05), with medium and significant effect sizes for the time taken for the studies. However, as can be seen in Table 8, there was no significant difference in the effect of AI on learners’ self-efficacy across different durations of the studies (Q = 1.872, *p* > 0.05).

## 4. Discussion

### 4.1. AI Can Effectively Promote Learners’ Self-Efficacy in Learning Contexts

In this study, we conducted a meta-analysis of 23 studies to analyze the effects of AI on learners’ self-efficacy in learning contexts; overall, we found that AI had a medium and significant positive effect on learners’ self-efficacy (0.758, *p* < 0.05). This finding is similar to a systematic literature review by Massaty et al. (2024), who found that AI can enhance students’ self-efficacy through multiple elements (e.g., interactive activities). Self-efficacy refers to the explicit beliefs or self-confidence that individuals hold about their abilities, namely, the expectations and judgments they make about their abilities during the learning process (Bandura, 1986). Prior learning experiences and curricular or instructional strategies are recognized as important factors influencing individual self-efficacy (Van Dinther et al., 2011). AI has been shown to have multiple roles in education, such as providing a personalized learning environment and timely feedback (Crompton & Burke, 2023). And these might further enhance students’ learning confidence whilst promoting their self-efficacy.

According to this result, the integration of AI into classroom can enhance students’ self-efficacy, which would contribute to their success in learning. Therefore, the role of AI to enhance a learner’s self-efficacy in learning contexts should not be underestimated. AI-related learning activities could help improve students’ self-efficacy and promote learning effectiveness. It is recommended that educators enhance the integration of AI in classrooms and design additional activities that promote student–AI interaction. Furthermore, due to the low numbers of experimental studies on the effects of AI on self-efficacy in learning contexts, the need for more experimental research in this domain is highlighted.

### 4.2. Moderating Effects of AI on Learners’ Self-Efficacy in Learning Contexts

#### 4.2.1. Learner Levels

In this study, analysis results revealed the impact of AI on the self-efficacy of learners at different levels (as shown in Table 2). AI had a significant positive impact regarding the self-efficacy of university students. Although AI also showed a positive impact on K-12, as well as other level learners, it was not significant. Meanwhile, statistical analysis (Q = 2.740, *p* > 0.05) did not show a significant difference in AI impact on self-efficacy at different learners’ levels, suggesting that the effect of AI on self-efficacy remains relatively consistent across different learners’ levels. Similarly to previous systematic review studies, AI was found to be mostly used in university education (Ng et al., 2023; Zhai et al., 2021). This suggests that although AI is effective at improving learner self-efficacy in university education, it might not be extensively utilized in K-12 education. This may be related to the numerous implementation issues of using AI at the K-12 level, such as age-appropriate educational content and infrastructure (Felix & Webb, 2024). More research needs to be undertaken to identify if and how educational material can be designed and used at the K-12 level to further facilitate students’ interactive learning with AI to enhance self-efficacy.

#### 4.2.2. Research Settings

The results showed that AI had a positive and significant effect on the self-efficacy of learners in the online context, as well as in the classroom context (as shown in Table 3). However, statistical analysis (Q = 0.289, *p* > 0.05) showed that there was no significant difference in the effect of AI on learners’ self-efficacy between the different research settings. This result suggests that the effect of AI on college students’ self-efficacy is relatively consistent across research settings. Our study also found that, among the articles included in this meta-analysis, most of the research contexts were based in a classroom, with only one research context set in an online context. This may be related to students’ trust and preference for people as teachers rather than AI agents in online courses (Spence et al., 2024); few courses have developed strategies to enable robust AI-integrated teaching and learning, few lecturers are willing to share their courses whilst still trialing, developing, and refining AI-focused coursework, and few lecturers are open to sharing course work that is unable to be formally assessed as true student work. With all these possibilities there is still much research to be undertaken in this area.

#### 4.2.3. Disciplines

The results of this study showed that the application of AI in four different disciplines—social sciences, medicine, humanities, and natural sciences—significantly increased learners’ self-efficacy (as shown in Table 4). But the effect value for learners of the engineering discipline was small and non-significant. Additionally, the statistical analysis (Q = 10.348, *p* < 0.05) showed that the effect of AI application on self-efficacy in different disciplines was significant, which indicated that it had the highest effect on self-efficacy for students of natural sciences and medical disciplines, and it had less of an effect on self-efficacy for humanities students. The results from the engineering disciplines was inconsistent with previous research findings. The study by Yilmaz and Karaoglan Yilmaz (2023) showed that ChatGPT significantly increased computer science students’ programming self-efficacy. This inconsistency may be due to the numerous interpretations of the term engineering or the plausibility that engineering disciplines may require students to utilize their personal understanding and practical experience, and, as AI gives direct answers, this may hinder the internalization process and thus deprive students of a source of self-efficacy (Yang et al., 2025).

We also found that of the 23 studies included in this meta-analysis, most of the articles conducted experimental research in the social disciplines, and the effect of AI on learners in these fields was large and significant. This may be due to the abundant learning resources and personalized learning aids available for AI integration (Ansari et al., 2024). This suggests AI may also enhance students’ self-confidence in accomplishing social discipline tasks, such as writing and speaking (A. Chen et al., 2025), whilst strengthening their self-efficacy. A previous study which investigated the use of AI in English language education found that student self-efficacy and speaking was significantly improved (Yıldız, 2024). This study found that the effect of AI on learners’ self-efficacy in different disciplines is differentiated, and it is suggested that educators need to consider the differences between disciplines when developing AI-integrated material.

#### 4.2.4. Type of AI Utilized

For the type of AI utilized in this study, the results have identified a significant positive impact on learners’ self-efficacy when the AI types utilized were intelligent learning environments and learning robots (as shown in Table 5). However, there was no significant effect on learners’ self-efficacy when the AI type utilized was an intelligent tutoring system. Statistical analysis showed (Q = 5.392, *p* > 0.05) that the difference between different AI application types on learners’ self-efficacy was not significant, which means that the effect of different AI application types on learners’ self-efficacy is relatively consistent. In this study, we summarized three types of applications of AI, namely, intelligent learning environments, educational robots, and intelligent tutoring systems from the included studies. Since AI is in its early stages and the full extent of AI types has not yet been developed and utilized to support education, educators need to consider the fit between AI types and teaching design. This is yet another research area for researchers.

#### 4.2.5. The Role of AI

The analysis of this study revealed a significant difference in the effect of the roles of different AI applications on learners’ self-efficacy (Q = 3.991, *p* < 0.05) (as shown in Table 6). Specifically, the highest effect value was found when the role of AI was as an intelligent learning tool, while the lowest effect value was found when the role of AI was multi-purpose and used as a mixed tool (i.e., both as an intelligent learning tool and an intelligent tutor). AI as an intelligent learning tool is mainly used by students, possibly to support their self-learning. The self-regulated learning of students is based on their thoughts, feelings, and behaviors that students then develop to achieve their personal goals (Alonso-Mencía et al., 2020; Zimmerman, 2000). Therefore, students can use AI to personalize their learning according to their own learning progress, which may enhance their self-confidence and self-efficacy in the process of task solving. Educators could give more consideration to students’ use of AI tools to promote greater self-confidence in the actual educational process.

#### 4.2.6. Length of Experimental Research Design

Initially, the study results revealed that all different time spans of the experiments have significant and positive effects as concerns AI, with regard to improving students’ self-efficacy (as shown in Table 7). This encourages educators to try to use AI and integrate AI into their learning classrooms, which may be able to help students increase their self-efficacy and self-confidence in learning. However, through further statistical analysis (Q = 1.872, *p* > 0.05), it was found that the difference in AI improving students’ self-efficacy across a range of time spans was not significant, indicating the effects were relatively consistent. Longer periods of technology learning can reduce the novelty effect and students’ interests (Sun & Zhou, 2024), while shorter periods of AI use may limit the time available to evaluate and identify change in student effectiveness or the time to develop skills and confidence with the new technology and delivery methods (Sun & Zhou, 2024).

### 4.3. Limitation and Future Studies

Although the present study provides valuable insights, and recommended future research was reported throughout the Discussion section, it has several limitations that should be acknowledged. Due to the inclusion criteria, only 23 papers were included in this study; these papers in the meta-analysis had relatively small sample sizes. And some relevant but not fully compliant papers may have been excluded. In addition, some subgroups in this study included fewer than five observations (N < 5), which may limit the precision of estimates in these categories. Although this study proposed relevant moderating variables based on existing studies, other potential moderating variables may have been overlooked. It is recommended that future studies incorporate more potential moderator variables to achieve more comprehensive and in-depth analyses. Meanwhile, the heterogeneity of this study is very high; the potential for unmeasured factors might account for a great deal of variation between included studies. Furthermore, although several approaches were used to evaluate publication bias in the present study, it remains essential for future research to implement more comprehensive methods to enhance the rigor of the assessment.

## 5. Conclusions

This study employed meta-analysis to answer two questions about the effects of AI on learners’ self-efficacy in educational contexts. In doing so, we found that (1) AI had a significant positive, medium-sized impact on learners’ self-efficacy, and (2) increases in learner self-efficacy were moderated by the context and purpose of AI use. Overall, this study revealed the effects of AI on self-efficacy in educational contexts. It analyzed the reasons that may influence the changes in the effects of AI on self-efficacy from the perspective of multiple moderating variables. This provides insights into the relationship between AI and self-efficacy and suggests ways to enhance the use of AI in education from the perspective of self-efficacy.

## Figures and Tables

**Table 1:** Inclusion and exclusion criteria.

Inclusion Criteria	Exclusion Criteria
(1) studies were from peer-reviewed journal articles or peer-reviewed conferences (2) the research topics investigated the effect of AI on students’ self-efficacy (3) the research situation was a learning context (4) the research was quantitative experimental or quasi-experimental studies conducted in English (5) the research data and outcomes were fully reported and provided the data needed to conduct a meta-analysis such as sample size (N), mean (M), standard deviation (SD), *t*-value, and *p*-value (6) the full text of the article was available (7) the article was published between January 2005 and February 2025	(1) studies were not from peer-reviewed journal articles or peer-reviewed conferences (2) the research topics paid no attention to the effect of AI on students’ self-efficacy (3) the research situation was not a learning context (4) the research was not quantitative experimental or quasi-experimental studies conducted in English (5) the research data and outcomes were not fully reported or did not provide the data needed to conduct a meta-analysis (e.g., sample size [N], mean [M], standard deviation [SD], *t*-value, or *p*-value) (6) the full-text of the article was not available (7) the article was not published between January 2005 and February 2025

Note. Statistical estimates extracted from the included studies, such as sample sizes, means, and standard deviations, were used to compute effect sizes.

**Table 2:** Overall effect size and homogeneity test results.

	Effect Size and 95% CI					Test of Null (2-Tail)		Heterogeneity				Tau-Squared		
Modal	N	Hedges’ g	SE	LL	UL	Z	*p*	Q	df	*p*	I^2^	Tau^2^	SE	Tau
Fixed	23	0.559	0.045	0.471	0.647	12.430	0.000	213.115	22	0.000	89.677	0.423	0.180	0.650
Random	23	0.758	0.147	0.470	1.045	5.162	0.000							

Note: CI = confidence interval; SE = standard error; LL = lower limit; and UL = upper limit.

**Table 3:** Effects of learner level on effect size.

				95% CI		Two-Tailed Test		
Moderator Variables	N	g	SE	LL	UL	Z	*p*	Group Differences
Learner level	23	0.745	0.144	0.463	1.028	5.176	0.000	Q = 2.740 *p* = 0.254
University education	16	0.813	0.161	0.497	1.129	5.037	0.000	
K-12 education	5	0.305	0.344	−0.369	0.980	0.887	0.375	
Others	2	1.568	0.853	−0.104	3.239	1.838	0.066	

Note: CI refers to confidence interval, g refers to effect size, SE refers to standard error, LL refers to lower limit, and UL refers to upper limit.

**Table 4:** Effects of research settings on effect size.

				95% CI		Two-Tailed Test		
Moderator Variables	N	g	SE	LL	UL	Z	*p*	Group Differences
Research Settings	23	0.715	0.119	0.481	0.948	5.998	0.000	Q = 0.289 *p* = 0.591
Classroom	22	0.768	0.155	0.464	1.072	4.951	0.000	
Online	1	0.638	0.186	0.273	1.002	3.429	0.001	

Note: CI refers to confidence interval, g refers to effect size, SE refers to standard error, LL refers to lower limit, and UL refers to upper limit. The category of online settings represents one study only and is included for exploratory purposes.

**Table 5:** Influence of disciplines on effect size.

				95% CI		Two-Tailed Test		
Moderator Variables	N	g	SE	LL	UL	Z	*p*	Group Differences
Disciplines	23	0.895	0.101	0.698	1.093	8.874	0.000	Q = 10.348 *p* = 0.035
Social sciences	9	0.894	0.248	0.407	1.381	3.597	0.000	
Medicine	5	1.013	0.271	0.482	1.543	3.741	0.000	
Engineering	4	0.060	0.449	−0.821	0.941	0.133	0.894	
Humanities	4	0.658	0.166	0.334	0.983	3.974	0.000	
Natural sciences	1	1.310	0.192	0.933	1.688	6.808	0.000	

Note: CI refers to confidence interval, g refers to effect size, SE refers to standard error, LL refers to lower limit, and UL refers to upper limit. The category of natural sciences represents one study only and is included for exploratory purposes.

**Table 6:** Effects of type of AI utilized on effect size.

				95% CI		Two-Tailed Test		
Moderator Variables	N	g	SE	LL	UL	Z	*p*	Group Differences
Type of AI utilized	23	0.574	0.079	0.421	0.728	7.315	0.000	Q = 5.392 *p* = 0.067
Intelligent learning environment	12	0.624	0.088	0.452	0.796	7.098	0.000	
Educational robot	9	1.185	0.447	0.308	2.062	2.648	0.008	
Intelligent tutoring system	2	0.233	0.190	−0.139	0.606	1.228	0.220	

Note: CI refers to confidence interval, g refers to effect size, SE refers to standard error, LL refers to lower limit, and UL refers to upper limit.

**Table 7:** Effects of the role of AI on effect size.

				95% CI		Two-Tailed Test		
Moderator Variables	N	g	SE	LL	UL	Z	*p*	Group Differences
Role of AI	23	0.544	0.089	0.369	0.719	6.082	0.000	Q = 3.991 *p* = 0.046
Intelligent learning tool	17	0.883	0.192	0.507	1.259	4.603	0.000	
Mixed	6	0.450	0.101	0.252	0.648	4.449	0.000	

Note: CI refers to confidence interval, g refers to effect size, SE refers to standard error, LL refers to lower limit, and UL refers to upper limit.

**Table 8:** Influence of the length of study on the effect size.

				95% CI		Two-Tailed Test		
Moderator Variables	N	g	SE	LL	UL	Z	*p*	Group Differences
Duration of experiment	23	0.667	0.121	0.430	0.904	5.513	0.000	Q = 1.872 *p* = 0.599
1–3 months	10	0.890	0.293	0.315	1.466	3.034	0.002	
<1 month	6	0.812	0.302	0.219	1.404	2.685	0.007	
>3 months	4	0.734	0.242	0.260	1.207	3.037	0.002	
Not clearly	3	0.481	0.187	0.115	0.848	2.574	0.010	

Note: CI refers to confidence interval, g refers to effect size, SE refers to standard error, LL refers to lower limit, and UL refers to upper limit.

## Data Availability

The original contributions presented in this study are included in the article. Further inquiries can be directed to the corresponding author.
